# The impacts of climate change on the biomechanics of animals

**DOI:** 10.1093/conphys/coz102

**Published:** 2020-01-13

**Authors:** Paolo Domenici, Frank Seebacher

**Affiliations:** IAS-CNR, Località Sa Mardini, Torregrande, Oristano, 09170 Italy; School of Life and Environmental Sciences A08, University of Sydney, Sydney, NSW 2006, Australia

**Keywords:** Climate change, Biomechanics, Locomotion, Animals

## Abstract

Anthropogenic climate change induces unprecedented variability in a broad range of environmental parameters. These changes will impact material properties and animal biomechanics, thereby affecting animal performance and persistence of populations. Climate change implies warming at the global level, and it may be accompanied by altered wind speeds, wave action, ocean circulation, acidification as well as increased frequency of hypoxic events. Together, these environmental drivers affect muscle function and neural control and thereby movement of animals such as bird migration and schooling behaviour of fish. Altered environmental conditions will also modify material properties of animals. For example, ocean acidification, particularly when coupled with increased temperatures, compromises calcified shells and skeletons of marine invertebrates and byssal threads of mussels. These biomechanical consequences can lead to population declines and disintegration of habitats. Integrating biomechanical research with ecology is instrumental in predicting the future responses of natural systems to climate change and the consequences for ecosystem services such as fisheries and ecotourism.

## Teaser

Here, we summarise how the dominant environmental changes associated with climate change can impact the biomechanics and movement of organisms.

## Introduction

Anthropogenic climate change will dominate ecosystem flux for the next decades if not centuries. The recent literature has given a much clearer picture of the expected impacts of global warming on physiological performance and thermal tolerance (Gunderson and Stillman, 2015; Sinclair *et al.*, 2016). However, the impacts of climate change are much broader, including changes in ocean circulation and acidification, increased frequency of hypoxic events, increased storm activity, altered rainfall patterns and flow regimes of freshwater streams and rivers (Aarnes *et al.*, 2017; Baynes *et al.*, 2018; Doney, 2010; Doney *et al.*, 2009). These changes will challenge the biomechanical performance of organisms.

Biomechanics is concerned with the function of biological systems and materials that underlie animal structure and movement, and environmentally induced changes in biomechanical properties will have far-reaching consequences for ecosystems (Higham *et al.*, 2016). For example, increased storm activity and wave action can have pronounced impacts on intertidal communities. The biomechanical stress of increased wave action can damage and remove macroalgae and invertebrates from rocky shores (Jensen and Denny, 2016; Jonsson *et al.*, 2006). Some organisms have biomaterial properties that increase resilience to high physical impacts (Blamires *et al.*, 2017; O'Donnell *et al.*, 2013). Nonetheless, environmental changes such as in temperature, physical impacts stemming from wave of wind action, and ocean acidification, interact and thereby increase the total impact on individuals and on interactions between species (Domenici *et al.*, 2017b; Kroeker *et al.*, 2016). Anthropogenic climate change represents a dominant selection pressure and one that is novel to most ecosystems. It is timely now to highlight the importance of biomechanics for climate change responses: the field has much to offer to increase understanding of the effects of climate change, but it has not yet made a concerted effort to address the new problems arising with the Anthropocene. The challenge lies in integrating large-scale biogeographic features of climate with small-scale physiological and biomechanical properties of individuals (Torossian *et al.*, 2016). Biomechanics can answer how environmental stressors affect the mechanical properties and the motion of organisms and can develop predictive mechanistic models (Gaylord *et al.*, 2001) that can significantly advance understanding of the ecological consequences of climate change. Below, we summarise how the dominant environmental changes associated with climate change can impact the biomechanics and movement of organisms.

### Musculoskeletal function and movement

**Figure 1:**
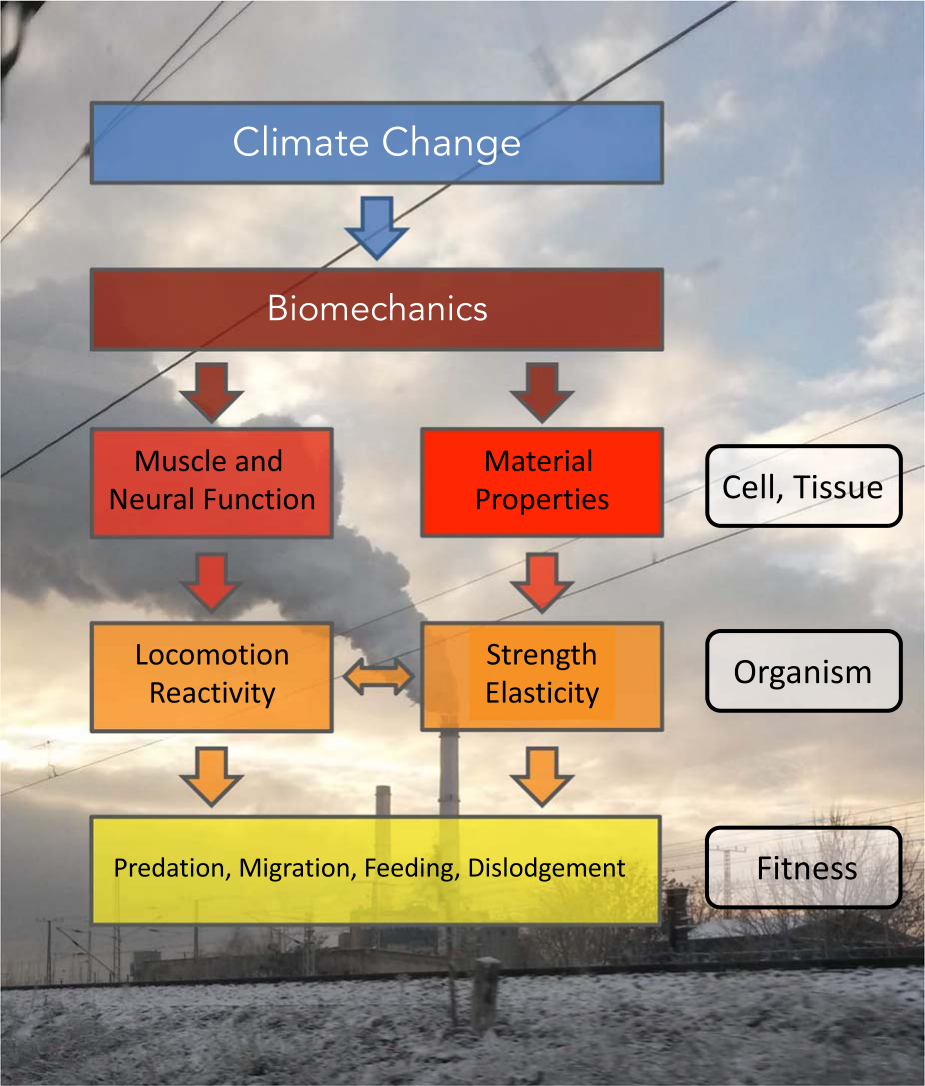
The effects of climate change on biomechanics and their potential consequences on the fitness of organisms. Conceptual diagram showing the effects of climate change at various biological levels. Climate change can affect: (i) muscle/neural function and (ii) material properties. In turn, these can affect the locomotor performance and reactivity of organism, as well as their strength and elasticity. These changes in organismal performance can lead to changes at the fitness level, in terms of vulnerability to predators and dislodgment, as well as feeding.

Animal movement depends on muscle and neural function, the cardiovascular system and metabolism, and the performance of these physiological systems is sensitive to environmental perturbations such as changes in temperature, pH, oxygen level, water flow and wind speed (Fig. 1). Climate change influences these parameters both by causing steady increases in temperature and decreases in oceanic pH, for example, and by increasing extreme climate events (van de Pol *et al.*, 2017). Environmental impacts on animal movement can compromise ecological networks such as food web structures and energy transfer between trophic levels by modulating predator–prey interactions (Gibert *et al.*, 2016).

The movement capacity of individuals and species will determine the relevant scale (from local to global) of environmental variability that affects performance and ultimately reproductive success. Migrating birds, for example, are exposed to local variation at their breeding and overwintering grounds and to global variation as a result of migration, and together these will determine reproductive success (Kentie *et al.*, 2018; Shaffer *et al.*, 2006). Altered wind and air currents modulate the biomechanics and energetics of bird flight and thereby affect survivorship and demographics (McCabe *et al.*, 2017; Sapir *et al.*, 2014; Weisshaupt *et al.*, 2017). In contrast, sessile organisms (e.g. intertidal mussels) are exposed only to local, but nonetheless complex, environmental variation (Choi *et al.*, 2019). Species with greater range of movement, such as migrating fish and birds, essentially undergo extensive bouts of endurance exercise while exposed to relatively large-scale climate variation. Endurance exercise is sensitive to muscle fatigue mediated by biomechanical constraints of intrinsic muscle function and metabolic energy supply (Allen *et al.*, 2008; Biewener, 2016). The combined effects of environmental parameters such as altered temperatures and flows (e.g. air and ocean currents, streams) can increase the cost of transport (Halsey, 2016; Seebacher *et al.*, 2016) and decrease muscle power output (James and Tallis, 2019), thereby compromising the movement objective (Martin *et al.*, 2015). Migrating salmon, for example, face reduced reproductive success because warmer water and altered flow conditions affect migration success (Eliason *et al.*, 2011; Fenkes *et al.*, 2016).

Climate change has already led to increased temperatures and increased wave heights in oceans (Aarnes *et al.*, 2017; Hemer *et al.*, 2013; Young *et al.*, 2011). Increased wave height results in greater impact force and may therefore lead to increased physiological demand on marine organisms (Bejarano *et al.*, 2017; Forrester *et al.*, 2016). At the same time, temperature changes alter muscle contractile function (James and Tallis, 2019), which can modify the impact of other drivers. Intertidal organisms are exposed to several environmental drivers concurrently (Choi *et al.*, 2019), and wave action is of particular importance because it can dislodge individuals and effectively remove them from the population (Jensen and Denny, 2016). One of the most important traits for survival of intertidal organisms is the adhesion strength to the substrate (Trussell *et al.*, 1993), and the foot muscle of snails, for example, has to produce greater force for the animal to stay in situ as wave action increases (Forrester *et al.*, 2016). At the same time, force needs to be produced longer if the increase in wave action is chronic. Increased wave action elicited a beneficial training effect on muscle by increasing tenacity, but increasing water temperature decreased endurance in an intertidal snail (*Nerita atramentosa*) (Clayman and Seebacher, 2019). Ocean warming would therefore negate the beneficial increase in tenacity that could render snails more resistant to acute impacts of wave action.

Warmer waters are often associated with low oxygen concentrations (Nudds *et al.*, 2020). Hypoxia and increasing temperature together influence metabolic scope and swimming energetics and kinematics (Claireaux and Lefrancois, 2007; Domenici *et al.*, 2013, Nudds *et al.*, 2020), which can lead to altered schooling behaviour in fish (Domenici *et al.*, 2017a). Increasing ocean temperature is often also associated with decreasing pH. Both as single and multiple stressors, acidification, temperature, and hypoxia can have pronounced effects on escape kinematics of fish that alter predator–prey interactions (Domenici *et al.*, 2019). Temperature often has a stronger effect than other environmental drivers (Domenici *et al.*, 2019; Nudds *et al.*, 2020). Overall, the climate-induced interaction between environmental drivers affects escape responses and swimming kinematics by decreasing muscle performance and interfering with brain and sensory function (Domenici *et al.*, 2019). Animals may compensate for changes in their environment, and acclimation can render individuals more resilient to environmental variability (Le Roy *et al.*, 2017). However, there is considerable variation between species in their capacity for plastic responses, which may determine spatial patterns of distribution of different species across climate gradients (Padilla *et al.*, 2019). One of the main challenges associated with predictions about the effect of global warming at the ecological level is that body temperature affects individual performance and that temperature varies through time within an individual and through space between individuals in a population (Denny, 2019). Theoretical work suggests that for any given individual this variation decreases performance and the range of viable temperatures (i.e. the range of temperatures within which animals can reproduce successfully). However, variation among individuals increases the viable range of the population (Denny, 2019). Clearly, investigating the effects of thermal history (as well as that of other main stressors) and plastic responses to variation at different time scales needs to be a priority in order to improve predictions of the effects of climate change on organisms.

In terrestrial environments, climate change can alter wind speeds (Young *et al.*, 2011), and altered wind speed patterns can influence migratory success in birds (Nourani *et al.*, 2017). Wind speed patterns can also disrupt predator–prey interaction by physically disturbing and dislodging individuals and by disrupting locomotion (Cherry and Barton, 2017). The terrestrial environment, including wind speed and temperature, can influence the energetic cost of transport (Chappell *et al.*, 2004; Halsey, 2016), which is a function of running kinematics (Kram and Taylor, 1990). Plastic responses to temperature can affect muscle and locomotor performance (James, 2013; James and Tallis, 2019; Padilla *et al.*, 2019), but probably not cost of transport (Jahn and Seebacher, 2019).

### Structural properties

Ocean acidification, in particular, has a strong negative effect on the structural properties of skeletons and shells of many marine invertebrates. Ocean acidification compromises calcification and growth twofold: the hypercapnia associated with increased CO_2_ causes an energetic constraint and allocation trade-off that curtails growth, and it reduces the calcium carbonate building blocks essential for calcification (Byrne and Fitzer, 2019). Increasing temperature can exacerbate this allocation trade-off. For example, shell strength of the mussel *Mytilus edulis* was negatively affected by increasing water temperatures which caused an allocation trade-off, where energy was directed away from shell formation to support temperature-induced increases in maintenance costs (Mackenzie *et al.*, 2014).

The combined effects of ocean acidification and increasing temperature are similar in bryozoans. Bryozoans are a phylum of aquatic invertebrates, which play an important ecological role as bioconstructors in shallow marine habitats. Their calcareous skeleton is quite different in structure and composition from that of other marine calcifying organisms such as molluscs. However, current and predicted future decreases in ocean pH and increases in temperature are detrimental for calcification of their skeleton, as well as growth rate and reproduction (Taylor *et al.*, 2014). In contrast, climate change-induced decreases in pH and warming had opposite effects in the tube built by the tube worm *Hydroides elegans*. Decreased pH weakened the tube and lowered resistance to predator attack, while increasing temperature increased mineral density and resistance to predator attack (Li *et al.*, 2016)

Corals are particularly sensitive to the combined changes in storm activity and wave action and ocean acidification. Branching corals are more susceptible to the combination of increased wave action and storm activity combined with sea-level rise than massive corals (Baldock *et al.*, 2014). Vulnerability of corals to wave stress is exacerbated particularly when corals lose condition and structural strength (Baldock *et al.*, 2014). These individual responses scale up to populations and may render populations vulnerable to decline and extinction. Acidification rendered populations of *Acropora hyacinthus* vulnerable to collapse by altering calcification rates and thereby compromising growth and skeletal strength (Madin *et al.*, 2012). These biomechanical impacts may be exacerbated by ocean warming, which can cause decreases in condition and coral bleaching (Hughes *et al.*, 2017; Mellin *et al.*, 2019).

Similar to corals, the interactions between environmental drivers have detrimental ecological impacts on marine mussels (Kroeker *et al.*, 2016). Mussels are common on temperate rocky shores around the world, where they form dense aggregations that provide essential habitat for ecological communities, and mussels are important as human food sources (Seitz *et al.*, 2013). Given their abundance and ecological and commercial importance, mussels have become a model system to study the interactions between individual responses to interacting environmental drivers and their consequences for population dynamics and persistence (Carrington *et al.*, 2015). Mussels attach to their substrate via byssal threads, and the biomechanical properties of the threads, and their sensitivity to environmental impacts such as ocean warming and acidification determines the survival of individuals and the persistence of populations (Newcomb *et al.*, 2019). Acidification and high temperature can affect mussel attachment strength and reduce mussel survival, though mussels may be more vulnerable to the negative effects of ocean warming than ocean acidification (Newcomb *et al*., 2019). However, single environmental drivers such as increased CO_2_ do not necessarily reduce attachment strength of the byssal thread (Dickey *et al.*, 2018). In addition to using multiple stressors, recent studies have made considerable advances in understanding the ecological relevance of climate change studies by using realistic fluctuating environmental conditions such as those likely to be experienced by organisms in nature (Frieder *et al.*, 2014; Gobler *et al.*, 2017; Jarrold *et al.*, 2017). Chan and Tong (2020) found that the responses of larval sea urchins (measured as growth of larval arms) were less pronounced when they were exposed to fluctuating low pH conditions, compared to a constant low pH. Therefore, particularly for larvae which are subject to varying environmental conditions throughout their dispersal, further work on the rate of physiological response to realistic (i.e. fluctuating) environmental conditions along the dispersive pathway is fundamental in order to predict the effects of climate change (Chan and Tong 2020).

Terrestrial ecosystems are of course also exposed to warming climates and storm activity. However, other environmental drivers such as rainfall patterns and drought and wind regimes have unique effects on non-aquatic habitats (Trenberth, 2011). The extended phenotype of terrestrial organisms may show plasticity in response to this environmental variation. Similar to the byssal thread of mussels, the strength and performance of other biological materials such as spider silk is influenced by environmental conditions (Blamires *et al.*, 2017). Climate change can affect spider webs by altering environmental variables that impact the physiological processes responsible for the production of silk. Temperature and humidity, in particular, can alter web architecture and performance (Blamires and Sellers, 2019).

## Conclusions

Climate change and its consequences are unavoidable now. A greater understanding of the impact of climate change on existing ecosystems will translate to better management and conservation of natural resources and biodiversity. The combined impacts of climate change on the biomechanics of animals are mainly due to effects on material properties and muscle/neural functions. These effects can have consequences for the fitness of the individual through their impacts on organismal strength and locomotor performance (Figure 1). Research in the area can be encapsulated by ecological biomechanics (ecomechanics), which provides a mechanistic framework linking environment-driven changes in the form and function of individuals, to community interactions and populations (Denny and Gaylord, 2010). This approach has been used successfully to model mussel biology with a focus on linking material properties to molecular and population responses (Carrington *et al.*, 2015). Biomechanical research is of particular interest here because it can link organismal traits to species interactions and community structure in the face of unprecedented environmental variability resulting from anthropogenic climate change (Gaylord *et al.*, 2019). It will be of particular importance that predictive models incorporate interactions between environmental drivers and natural fluctuations in those drivers.

In a broader context, the synthesis provided here can be applied to practical problems. The natural environment provides essential services to human societies. Ecological biomechanics can facilitate understanding of the mechanistic basis underlying many practical problems associated with climate change and can be a key element for developing relevant management and conservation strategies. For example, the sustainability of fisheries around the world depends on prediction of large-scale movements of commercial species. Movement of fish schools will depend on prevailing thermal conditions and sufficient food supply, which in turn will be affected by ocean acidification and ocean currents. These relationships, and others associated with aquaculture, farming, ecotourism, etc., will continue to change, and there is an urgent need for better mechanistic basis that can help predict the direction and magnitude of that change.

## Funding

F.S. was supported by the Australian Research Council Discovery Grant DP180103036. P.D. was supported by European Union’s Horizon 2020 research and innovation program under the grant agreement No. 773713 (PANDORA).
